# Chinese cognitive processing of ToM: Distinctions in understanding the mental states of self, close others, and strangers

**DOI:** 10.3389/fpsyg.2023.895545

**Published:** 2023-02-06

**Authors:** Yuanqing Wang, Xiaojing Yuan

**Affiliations:** ^1^School of Psychology, Nanjing Normal University, Nanjing, China; ^2^School of Teacher Education, Nanjing Xiaozhuang University, Nanjing, China

**Keywords:** theory of mind, culture, close others, strangers, perspective-taking

## Abstract

Previous studies showed that people differ in attributing mental states to themselves and in understanding the mental states of others, but have not explored the differences when people attribute mental states to others at different social distances. The present study adds a ‘close other’ condition to the Self/Other differentiation paradigm to explore the potential differences in attributing mental states to others with different social distances. It emerged that the time required to reflect on one’s self mental state is shortest in mental state attribution, longer when comprehending the mental state of close others, and longest for strangers. This result indicates that Chinese participants distinguish between close others and strangers when performing perspective-taking. When the perspective-shifting of belief-attribution is performed, a beforehand processing of information about close others or strangers does not interfere with the processing of information from oneself subsequently. However, when the information processed in the previous stage cannot be used for subsequent processing, it interferes with the processing of information from close others or strangers in the later stage. The lower the degree of automated processing of pre-processed information, the greater the interference effect produced. This finding indicated that processing the self mental state is automatically activated, but comprehending the mental state of others is not. The comprehension of others’ mental states occurs only when required by the task and it entails more cognitive resources to process and maintain.

## 1. Introduction

Theory of Mind (ToM) is defined as the ability to attribute a mental state to oneself and to others (Baron-Cohen and Wheelwright, 2004). This mental ability is the basis of information exchange in social interaction. The modular structure concept of ToM holds that ToM involves distinct components that encompass specific, distinct mental processes (e.g., Decety and Sommerville, 2003; Harari et al., 2010). As evidenced by some studies, reflecting on one’s own mental state and understanding the mental states of others may involve different processing modes (Jardri et al., 2011; van der Weiden et al., 2015).

Nonetheless, some researchers have suggested that social distance from the self may influence perspective-taking about others (e.g., Lee and Atance, 2016; Farrar and Ostojić, 2018). There are also cultural differences in the social distance between self and others. Cross-cultural psychology shows that, in Western society, the construct of self is independent and there is a clear boundary between self and others, while in East Asian society, individual self-construal is a dependent self that depends on others and complex social networks (Markus and Kitayama, 1991). Studies have shown that Chinese people’s concept of self includes specific, very close relatives. For example, prior research found that different from Western people, Chinese people are inclined to integrate the representation of a close other into the representation of self, but not so regarding a stranger (e.g., Han and Zhang, 2013; Wang et al., 2017). This may make it easier for Chinese people to understand the mental states of close others, i.e., relatives and friends, than to consider mental states of strangers. Thus, exploring the differences in how people understand the mental states of others at different social distances can be considered to be beneficial in cultures where the psychological distance between self and others is more pronounced.

Most current research has focused on discussing the differences in perspective-taking between the self and others while failing to explore whether there are differences in attributing mental states of others who are at different social distances. Hence, this study aims to investigate whether people use different processing modes to practice theory of mind for their own mental state and for the mental state of close others and strangers in the Chinese cultural background.

### 1.1. Different ToM processing patterns between self and other

Whether understanding oneself and understanding others employ different processing modes when ToM abilities are applied has given rise to extensive debate. Some developmental psychologists have suggested that ToM involves a single mechanism, and there is no clear difference between the development of self-and other-oriented mental states (Wellman et al., 2001). However, some psychologists have argued that, if the ability to apply ToM cannot be fully developed, or is interrupted due to illness or injury, the ToM of the self and that of others may involve different processing modes. For example, individuals with autism spectrum disorder are prone to exhibit egocentric behaviors in ToM tasks, and these patients generally have low social cognitive ability in comprehending the mental states of others (Hutchins et al., 2011). This supports the distinction between ‘self’ and ‘other’ in the application of ToM. Neuropathological studies have also found that adult patients with right-sided frontotemporal brain injury have deficits in their ability to inhibit ‘self-perspective’ while retaining the ability to infer the perspective of others (Samson et al., 2005). Researchers concluded that both the right temporo-parietal junction and brain areas associated with the human mirror neuron system are likely to critically influence self-other distinction. The appropriate degree of self-other distinction will vary according to the exact social situation, e.g., affective empathy may involve low self-other distinction, whereas understanding false belief requires higher self-other distinction (Eddy, 2022). These studies demonstrate that there is a difference between the processing of the ‘self’ perspective and the processing of the ‘other’ perspective.

Previous studies have shown that there is a difference between the ‘self’ and ‘others’ when considering and attributing mental states, and there is still some controversy about the degree to which the ‘other’ perspective is automatically processed. One opinion holds that the views and beliefs of others are automatically processed along with the ‘self’ perspective no matter in adults or children (Kovács et al., 2010), while another opinion suggests that the ‘other’ perspective is processed only when required by the task (Back and Apperly, 2010). Some evidence has shown that the ToM capability is susceptible to the executive function (EF) capability in adults, but EF capabilities may be invoked only when the motivation and cognitive resource is sufficient (eg., Schneider et al., 2012; Low et al., 2016; Cane et al., 2017). For example, Schneider et al. (2012) employed a dual-task method to investigate the effects of executive load on eye movements in the implicit Sally-Anne false belief task. The results revealed that even the most low level processing of beliefs appears to reflect an operation that can be limited by other capacities, such as working memory. Other research also suggested the indirect constraints on reasoning the belief about others imposed by EF. For instance, Brown-Schmidt (2009) found that it is more natural and less cognitively costly for adults to adopt an egocentric frame of reference than to deliberately consider the perspectives of others during conversation. Vice versa, the heavier cognitive load usually leads to more egocentric bias (Epley et al., 2004; Mckinnon and Moscovitch, 2007). Therefore, the processing of non-automatic ToM is bound to be affected by cognitive resources.

The Self/Other Differentiation task was designed to assess the ability of healthy adults to apply theory of mind (Bradford et al., 2018). The task is a novel task based on the standard false belief paradigm, in which belief states are created from either the other-perspective or the self-perspective, and remained matched in structure and formation, enabling participants to explore their own or others’ belief states. This task consisted of the Dilemma stage and the Probe stage. The Dilemma stage was presented first. The main purpose of this stage is to identify the mental state. The Probe stage is mainly used to evaluate the mental state attribution abilities of participants (Bradford et al., 2015). When using this task, Bradford et al. (2015) found that people consume more time when attributing beliefs to other people as opposed to recognizing and attributing beliefs to oneself.

### 1.2. Self/other perspective-taking: From a cultural perspective

There are scholars who have proposed that mental state attribution varies across different cultures. Prior research has found that people from interdependent cultures are more efficient (faster\more accurate) when attributing others’ mental states than those from independent cultures (e.g., Wu and Keysar, 2007; Kessler et al., 2014). Wu and Keysar (2007) compared the performance of Chinese and Americans in the above regard by conducting an eye-tracking study using a visual perspective-taking task. It was found that Chinese participants were distracted to a lesser extent by their own, personal perspective, made fewer mistakes while evaluating the intentions of another person, and were better at solving problems related to perspective-taking as compared to Americans.

Scholars contend that Western culture is often considered individualistic, while East Asian culture is considered collectivistic. Collectivistic cultures exhibit interdependence and feature self-construal that incorporates the significance of relationships and social obligations (Triandis et al., 1988). On the other hand, people of individualistic cultures endeavor toward independence and have concepts of self-defined by personal aspirations and accomplishments (Shweder and Bourne, 1982). Therefore, rather than focusing on the mental states of other people, members from the individualistic cultures tend to focus more on their own states of mind. Contrariwise, it is on the mental states of others rather than their own that members of collectivistic cultures focus on more.

However, several recent researchers have found inconsistent results regarding whether the influence of culture is predominant. For example, Wang et al. (2019) compared the performance of British (independent) and Taiwanese (interdependent) participants in two perspective-taking tasks. They reported no significant difference in both altercentric and egocentric interference between the two cultural groups. Similarly, Bradford et al. (2018) did not find a cultural influence on the performance of participants from Western and Chinese cultures in a computerized false-belief task. These researchers argue that equivalent performance in differentiating the self from the other perspective across cultures is due to basic processing mechanisms that underlie perspective-taking likely being shared among different cultures. It is suggested that the process of differentiating ‘self” from ‘other’ may reflect the fact that it is a core component of the ToM mechanism applied in similar ways across different cultures. Researchers believe that the processing mechanism that differentiate ‘self’ from ‘other’ is of such strong influence that the influence of culture on the attribution of mental state is relatively weak. In order to resolve this controversy over different performance results among different cultures, it is necessary to understand the boundaries between the self and other from a cultural perspective.

The concept of ‘others’ is closely related to social culture. In collectivism, researchers often make a more detailed division of the concept of ‘others’. For example, in the study of Chinese social cognition, researchers usually further divided the concept of ‘others’ into ‘close others’ (such as mothers) and strangers (Zhu and Zhang, 2002; Sui et al., 2012, 2014). The Chinese culture also emphasizes the connection between the self and others (Markus and Kitayama, 2010). Researchers found that Chinese who hold interdependent selves have a certain degree of overlap with their close family members and friends, and the psychological distance between them can be regarded as ‘zero’ (Wang et al., 2011). Cultural neurological studies have shown no significant differences in the intensity of activity in specific brain regions between Chinese representations of self and of mother, whereas Westerners separate the two representations in terms of the intensity of activity in specific brain regions. This suggests that, to some extent and unlike Westerners, the Chinese self includes close others such as mothers (e.g., Vanderwal et al., 2008; Wang et al., 2017). Ding et al. (2020) performed an ERP study and provided evidence for the inclusion of a ‘mother’ component in the Chinese concept of self as seen from the perspective of face emotional processing. These studies suggest that the Chinese self shares many similarities in mental representations with close others, and that the two may be shared representations, i.e., have the same neural coding in perceptual and behavioral processes.

The East Asian cultural environment’s emphasis on inter-individual connections has led to the integration of the self with close others, such as the mother, in neural representations. This may make Chinese people perform better (faster\more accurate) when attributing mental states to close others than to strangers, attributing their own mental states. In their experiment, Varnum et al. (2014) found that the degree of activation of the bilateral ventral striatum was more influenced by relational closeness after priming the interdependent self, suggesting that interdependent selves are more likely to have positive empathy for close others. Research on self-other overlap has found that the higher the degree of overlap between self and other, the more likely an individual is to adopt a perspective regarding others (Myers et al., 2014). Research in the field of decision making also shows that the closer to others in psychological distance that one is, the greater the agreement in decisions made for others are to those made for oneself (Liviatan et al., 2008).

Given the clear distinction between close others and strangers in the Chinese cultural context, we prefer to argue that the influence of culture on perspective-taking is mainly reflected in the different distances between ‘close others’ and strangers, with the ‘self’ as the anchor point. The proximity of ‘close others’ and ‘strangers’ to the self has the potential to influence the cognitive resources people consume when adopting the perspectives of others, and thus the efficiency of perspective-taking.

Taken together with previous research, we hypothesized that people may employ different processing patterns when they consider their own mental state versus understanding others. In this context, Chinese people also differ in their adoption between the ‘close other’ and ‘stranger’ perspectives because, in the Chinese cultural context, the self-concept includes the close others, i.e., the distance between close others and the self is very small. Therefore, we proposed two hypotheses:

- In the Self/Other Differentiation task, Chinese people will respond fastest to perspectives of self, and they will perform better when adopting perspectives from close others than strangers.
- When the information processed in the previous stage cannot be used for subsequent processing, it will interfere with the processing of information from close others or strangers in the later stage. The lower the degree of automated processing of pre-processed information, the greater the interference effect will be produced.

## 2. Materials and methods

### 2.1. Participants

Seventy students (42 females; mean age = 22, SD = 3.51) were recruited from Nanjing Normal University. All participants were in good health with normal or corrected-to-normal vision and had never participated in similar studies. Each participant received 10 yuan when they finished the experiment. Participants gave informed consent before the experiment and this study was approved by the Ethics Committee of the Nanjing Normal University.

### 2.2. Measures

The present study adds a “close other” condition to the Self/Other Differentiation task developed by Bradford et al. (2015) to explore the potential differences in attributing mental states to others with different social distances. The Self/Other Differentiation task has previously been used with healthy adult samples, demonstrating that in these samples, self-oriented processing is much more efficient (faster and more accurate) than other-oriented processing (Bradford et al., 2018). Based on Bradford et al. (2015), images were selected from the Bank of Standardized Stimuli (Brodeur et al., 2010) and the Amsterdam Library of Object Images (Geusebroek et al., 2005) to create the stimulus presentation materials for the experimental task. The ‘close other’ consisted of immediate family members of participants, including their father, mother, elder brother/sister, and the younger brother/sister. The ‘stranger’ consisted of six fictitious names (Linda, Nathan, Susan, Roy, Anna, and Tom) created by the experimenter.

### 2.3. Procedure

The experiment ran on computers with 17-inch screens and used E-prime 2.0 software. The Self/Other Differentiation task consisted of three stages, using containers and objects common to daily life as materials. The Dilemma stage was presented first. The main purpose of this stage is to identify the mental state. Participants were required to select one container image among three under either a self-oriented or other-oriented perspective to find a specific object. Then came the contents revelation stage. Content in the container that had been selected in the Dilemma stage was shown in this stage. The content could be either anticipated (e.g., a computer in the computer bag) or unanticipated (e.g., a water cup in the camera bag). The last stage is called the Probe stage. This stage is mainly used to evaluate the mental state attribution abilities of participants. Participants were told to pick out what they (self-oriented) or another individual (other-oriented) thought was inside before looking in the container.

**Figure 1:**
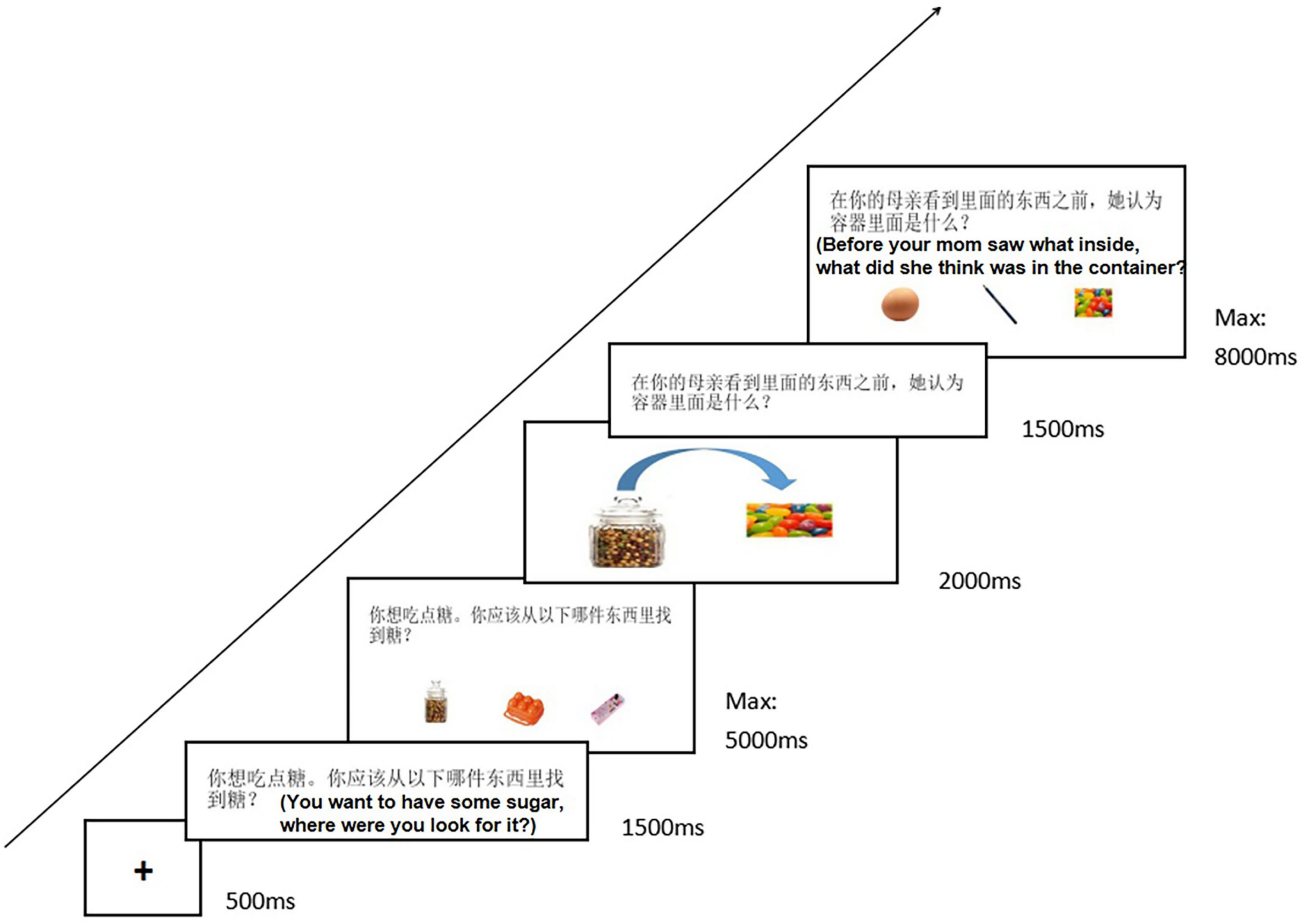
Illustration of the different stages of a single trial in the Self/Other Differentiation Task: the Dilemma Stage (‘Self’, ‘Close other’, or ‘stranger’), Consents Revelation Stage (Excepted or Unexpected), and Probe Stage (‘Self’, or ‘stranger’).

The specific process was as follows: Each trial consists of six steps (see Figure 1 for the detailed descriptions and sequence of a single trial). First, a centrally located fixation point was displayed for up to 500 ms. After 500 ms of a blank screen, questions in the Dilemma stage were presented for 1,500 ms without images (e.g., You need to use a computer for your work, where would you look for it?). For the Dilemma task, the options for the image answer (e.g., three containers: computer bag, wardrobe, refrigerator) were subsequently shown for up to 5,000 ms. Participants were required to press the key to select the options. If participants made a wrong choice (e.g., wardrobe) or if they did not respond within the time limit, a red ‘**×**’ appeared on the screen. Then came the fourth step, the expected/unexpected content in the selected container was displayed for 2000 ms (e.g., computers/snacks in the computer bag). Next, the Probe question appeared without any images and was presented on the screen for 1,500 ms (e.g., What do you think is inside the container before you see it?). Finally, the Probe question and images of contents in three containers were presented for a maximum of 8,000 ms. Participants were required to choose the correct answer (e.g., computer) within the time limit. If there was no response for more than 8,000 ms, a red message ‘response too slow’ appeared on the screen. The trial ended at this point. The sentence length of the Dilemma question in each trial was no more than 25 words, as was the length of the Probe question sentence.

There were six practice trials and 72 formal test trials in the task. Formal test trials were presented in a counterbalanced form, with each trial comprised of the aforementioned stages. Both the Dilemma stage and the Probe stage contains three conditions: ‘self’ perspective, ‘close other’ perspective, and ‘strangers’ perspective, while the contents revelation stage contains two conditions: expected contents and unexpected contents.

## 3. Results

Previous researchers regarded the response time of the Dilemma stage and Probe stage as two separate times and suggested that they should be analyzed separately (Bradford et al., 2015). We adopted the same analysis method as in previous studies, i.e., if participants respond incorrectly during the Dilemma stage, their data would be excluded from the response time of the Dilemma stage. However, if the participants answered correctly at the Probe stage, the response time data would be included in the Probe stage analysis. If the mean response time of one participant differed by more than three standard deviations from the mean response time of all participants, all data for that participant were intended to be excluded. No outliers were observed in this study; all data of participants were thus kept for analysis.

### 3.1. Dilemma stage

For the Dilemma stage, there are three perspective types: ‘self’ perspective, ‘close other’ perspective and ‘stranger’ perspective. A repeated ANOVA was conducted with perspective types (self vs. close other vs. stranger) as independent variables, with response times as a dependent variable (Descriptive statistics for the response time can be found in Table 1). A significant main effect of perspective type was detected, *F* (2, 138) = 47.44, *p* < 0.01, *η^2^* = 0.41, indicating that participants differed in RTs between the three perspective types. Post-hoc tests revealed that RTs in the ‘self’ perspective (*M* = 1,279 ms) were significantly faster compared with RTs for the ‘close other’ perspective (*M* = 1,417 ms, *p* < 0.01) and ‘stranger’ perspective (*M* = 1,490 ms, *p* < 0.01; see Table 1). The results for the Dilemma stage demonstrated that participants responded fastest when carrying out self-oriented reasoning, compared to the close other-oriented or the stranger-oriented reasoning. Moreover, participants responded faster in the ‘close other’ perspective than in the ‘stranger’ perspective (*p* < 0.01).

**Table 1:** Descriptive statistics for the response time of dilemma stage (*M* ± SD).

	Self perspective	Close other perspective	Stranger perspective
Response time(ms)	1,279 ± 270	1,417 ± 274	1,490 ± 280

### 3.2. Probe stage

The relationship between Dilemma type and Probe type was considered in the RTs analysis of the Probe questions, leading to a factor called ‘perspective shift’. In trials without perspective-shifting, both the Dilemma and Probe stage involved three perspectives, i.e., ‘self’, ‘close other’, and ‘stranger’. In trials with a shift in perspective between the two stages (Dilemma stage and Probe stage), which may be from the ‘self’ in the Dilemma stage to the ‘close other’ or ‘stranger’ in the Probe stage, or from the ‘close other’ or ‘stranger’ in the Dilemma stage to the ‘self’ in the Probe stage. In addition, the contents revelation stage contains expected contents and unexpected contents which lead to the true/false belief trials. Previous studies have shown that the main effect of true/false beliefs did not reach the significance level (Bradford et al., 2015, 2018). Therefore, no further distinction was made between true/false belief trials in the present study (descriptive statistics are shown in Table 2).

**Table 2:** Descriptive statistics for the response time of probe stage (*M* ± SD).

	Self dilemma	Close other dilemma	Stranger dilemma
Self probe	924±334.7	931±339.3	947±368.6
Close other probe	1,004±493.1	904±383.9	1,185±430.1
Stranger probe	1,006±420.0	1,158±460.8	954±410.1

To assess whether there were differences in the attribution of mental states by participants when processing the ‘self’ versus ‘close other’ versus ‘stranger’, a 3 (Dilemma stage perspective: self vs. close other vs. stranger) × 3 (Probe stage perspective: self vs. close other vs. stranger) repeated-measures ANOVA was performed with RTs of the Probe stage as the dependent variables (see Table 3). A significant main effect of the Probe stage perspective was detected, *F*(2, 138) = 5.46, *p* < 0.01, *η^2^* = 0.07, while there was no significant main effect of the Dilemma stage perspective, *F*(2, 138) =1.36, *p* > 0.05. Post-hoc tests revealed that RTs of self-oriented Probes (*M* = 934 ms) were significantly faster compared with RTs of ‘close other’ oriented (*M* = 1,031 ms, *p* < 0.01) and ‘stranger’ perspective (*M* = 1,039 ms, *p* < 0.01). There was no statistically significant difference in RTs between the ‘close other’ perspective and the stranger’ perspective (*p* > 0.05). The results suggested that self-information processing is automatic. The processing of information about self in the subsequent stage is unaffected by the information processed in the previous stage, whether it came from close friends or total strangers.

**Table 3:** Repeated-measures ANOVA results.

Variable	SS	Df	MS	*F*	*p*
Dilemma stage(A)	272978.235	2	713545.054	5.464	0.005
Probe stage(B)	1427090.109	2	136489.118	1.364	0.259
A × B	4152851.036	4	1038212.759	9.520	0.000
Error	30098655.741	276	109053.101		
Total	35952919.381	284			

A significant interaction effect between the Dilemma stage perspective and the Probe stage perspective was detected, *F* (4, 276) = 9.52, *p* < 0.01, *η^2^* = 0.12. Simple effect analysis revealed that there was no significant difference in three perspective-shifting conditions, i.e., self-to-self (*M* = 924 ms), close other-to-self (*M* = 931 ms), and stranger-to-self (*M* = 947 ms). Moreover, RTs for close other-to-close other (*M* = 904 ms) was faster than for self-to-close other (*M* = 1,004 ms, *p* < 0.05), while RTs for self-to-close other (*M* = 1,004 ms) was faster than stranger-to close other (*M* = 1,185 ms, *p* < 0.01). Similarly, RTs for stranger-to-stranger (*M* = 954 ms)was faster than for self-to-stranger (*M* = 1,006 ms, *p* < 0.05), while RTs for self-to-stranger (*M* = 1,006 ms) was faster than close other-to-stranger (*M* = 1,158 ms, *p* < 0.01). These results suggest that understanding the mental states of close others and strangers is task-driven and only happens when necessary. Additionally, information from the earlier stage is preserved during the two-stage perspective-shift task. As a result, this information is available for further processing when there is no perspective shift in the later stage, making it easier for participants to understand others’ perspectives in this stage. However, when a perspective shift occurs in a later stage, information from the earlier stage cannot be used for subsequent processing. The more cognitive resources are required to comprehend new information, the less conducive it is to subsequent understanding other’s perspectives.

## 4. Discussion

The current study explored the distinction between considering one’s own mental state and understanding the mental state of others. The results of the Dilemma stage showed a distinct behavioral differentiation between the processes required for attribution of mental states to close others and strangers. Although participants respond fastest when attributing mental states to themselves, they respond faster when taking perspectives from close others than from strangers. This result supports the hypothesis that Chinese participants do distinguish between close others and strangers when performing perspective-taking. This result is consistent with findings from cultural psychology related to Chinese people’s mental processing of self-versus others, i.e., the Chinese self-construal is inclusive of close others.

This result is also consistent with the viewpoint of a two-stage processing system. Specifically, the unconscious, automatic activation of self-concept occurs first, with egocentric anchoring of the self-mental state, followed by trying to comprehend others’ mental state (if there are differences between the self and the others’ mental state, an egocentric mentality is overcome in this stage). That is to say, when people adopt others’ perspectives, they do not put their own perspectives aside, but rather consider them a starting point or an anchor point for judgment (Epley et al., 2004). Therefore, the higher the degree of self-other overlap, the easier it is for individuals to adopt perspectives from others (Myers et al., 2014).

Prior research has found that social distance may influence the extent of egocentric bias in ToM processing (Farrar and Ostojić, 2018). In Chinese culture, where interdependence, relatedness, and harmony are emphasized and pursued (Fiske et al., 1998), people have a tendency to describe the self as being embedded in interpersonal and social contexts (Markus et al., 1997). Therefore, there is a certain amount of overlap among Chinese people with regard to close family members and friends, with psychological distancing among them being virtually eliminated. This result reflects the phenomenon that people have a dominant processing effect on the information processing of people closely related to them. This dominant processing effect phenomenon has also been found in previous cross-cultural studies on self-face recognition. In this case, Chinese participants showed a typical ‘Boss-Effect’ when viewing one’s own face or a supervisor’s face, i.e., they recognized their supervisor’s face faster than their own face. American participants on the other hand did not show this phenomenon (Liew et al., 2011). Thus, the dominance effect on incorporating the kin perspective may reflect the fact that, among individuals in the Chinese cultural context, relatives are included in the individual’s self-concept and thus implicitly or explicitly attract more attentional resources during information processing, generating a processing advantage.

In the Probe stage, there is no significant difference under the three conditions of self-self, close others-self, and strangers-self. This result indicates that when information from either close others or strangers, is processed in the former stage, it does not affect the processing of information about oneself in the later stage. However, when information from oneself was processed in the previous stage, it interfered with the processing of information from both close others and strangers in the later stage. The results show that under the condition of close others–close others, the response speed of participants was faster than under the condition of self–close others. Similarly, the response speed of participants under the condition of strangers–strangers is faster than that under the condition of self–strangers. This finding is consistent with the results of a study done by Bradford et al. (2015), suggesting that the processing of self-information is an automated process and that understanding the mental states of close others and strangers is task-driven, requiring additional cognitive processing and occurring only with explicit cues. Moreover, in perspective-shifting tasks, the information generated at the previous stage of processing is maintained. However, if the information is not used for subsequent processing, it may occupy cognitive resources, which is not conducive to the subsequent adoption of other people’s perspectives. That is, when others’ perspectives are considered alone, social distance does influence people’s attributions of mental states to others who are of varying degrees of intimacy. However, when there is a shift in perspective from self to others, the effect of egocentric is so strong that it weakens the influence of social distance.

Our study advances previous research in several ways. First, based on the previous experiment (Bradford et al., 2015), we further subdivided the ‘other’ perspective into ‘close other’ and ‘stranger’ perspectives, and explored whether the belief attribution of the three perspectives of self, close others, and strangers is different. On the one hand, with the introduction of the close other and stranger variables, we replicated previous findings that the ‘self’ may serve as a source of understanding the ‘other. Accepting the perspective of others, even close others with a high degree of overlap with the self, requires more cognitive effort than recalling and reflecting on self-oriented mental states. On the other hand, our findings suggest that people in the Chinese cultural context do differ in their adoption of close others’ perspectives and in their consideration of strangers’ perspectives. This finding supports the idea that the interdependent self has an influence on people’s mental processing of self and others in Chinese culture. Moreover, we further divided others into close others and strangers for comparison in our study, providing a new direction for cross-cultural psychological research on perspective-taking. Previous cross-cultural studies exploring the perspective-taking of self and others have typically compared the influence of different cultures on the perspective-taking of self and others in a general way, without a fine distinction between close others and strangers. This may also be a reason for the inconsistent findings in previous studies. People’s perspectives on intimates and strangers may differ across cultures.

Our study has limitations. First, we included ‘close others’ and ‘strangers’ because of the potential influence of culture on people’s psychological processing of self, close others, and strangers, but in this study, we did not recruit people from Western cultures as participants for comparison. Thus, we cannot assert that the same phenomenon does not exist in Western samples. Especially previous studies found that western and eastern samples are not restricted to being only independent or interdependent (Jiang et al., 2019). For example, For example, studies found that in some cases Americans are not less collectivistic than East Asians (Oyserman et al., 2002). Therefore, the results of this study need to be interpreted with caution. In the case of this study, interdependent culture should be considered a potential explanation for the results and not a definitive cause. Future cross-cultural research needs to examine whether participants from multiple cultures differ in their perspectives on the adoption of close others and strangers. Moreover, it should be noted that the innovative nature of the experimental paradigm in this study is double-edged. Since we did not conduct any pilot study, multiple tests in more different regions and types of populations are needed in the future to verify the external validity of the task and improve the generalizability of the results. Second, this study was conducted on a university campus where the participants were highly educated young adults. The sample was therefore underrepresented and inadequately diverse; future testing of the generalizability of these current findings across a larger age range will also be needed. In addition, some researchers argue that Chinese people are not culturally homogeneous and that there may be differences in the cultural influences on people from different regions. Wei and Wang (2020), for example, suggested that people who inhabit the traditional wheat farming regions of northern China exhibit more typical individualistic cultural behaviors compared to Chinese in the south. In turn, differences between regions may influence peoples’ mental processing of others of different social relationships. It will be necessary for the future to compare whether Chinese people from different regions differ in their adoption of the perspectives of others with different social relationships. Third, there is a limitation on the materials chosen for the experiments. Chinese translations of some foreign names were employed in the experimental task to represent “stranger,” which could cause some participants to classify these names as outgroups in addition to considering them strangers, adding extra confounding variables. Future research should use other naming strategies to preclude the potential effects of such confounding variables. Fourth, our study focused only on whether there is a difference in people’s mental processing concerning the perspective of people of different social relationships. However, theory of mind includes not only perspective-taking but also other affective components. Future research could explore whether there are distinguishable patterns of processing between the self and others in different social relationships in other theory of mind tasks. Fifth, in order to compare the effects of the “perspective shift” directly, no further distinction was made between true/false beliefs in the experimental task in the present study. This may ignore the potential moderating effect of belief type. In future research, there is a need to find more appropriate analysis methods that incorporate belief types. In addition, our findings are limited by the nature of behavioral experiments and thus cannot fully clarify the differences in the specific mechanisms by which people attribute themselves and understand others. Further neuroimaging studies are needed in the future to light on the specific processing mechanisms.

## Data availability statement

The original contributions presented in the study are included in the article/supplementary material, further inquiries can be directed to the corresponding author.

## Ethics statement

The studies involving human participants were reviewed and approved by the Ethics Board of Nanjing Normal University. The patients/participants provided their written informed consent to participate in this study.

## Author contributions

XY and YW contributed to the conception and design of the study. XY and YW collected and analyzed the data. YW wrote the first draft of the manuscript. All authors contributed to manuscript revision, read, and approved the submitted version. XY supervised the study.

## Conflict of interest

The authors declare that the research was conducted in the absence of any commercial or financial relationships that could be construed as a potential conflict of interest.

## Publisher’s note

All claims expressed in this article are solely those of the authors and do not necessarily represent those of their affiliated organizations, or those of the publisher, the editors and the reviewers. Any product that may be evaluated in this article, or claim that may be made by its manufacturer, is not guaranteed or endorsed by the publisher.
